# Heparan Sulfate: A Ubiquitous Glycosaminoglycan with Multiple Roles in Immunity

**DOI:** 10.3389/fimmu.2013.00470

**Published:** 2013-12-18

**Authors:** David Anak Simon Davis, Christopher R. Parish

**Affiliations:** ^1^Cancer and Vascular Biology Group, Department of Immunology, The John Curtin School of Medical Research, Australian National University, Canberra, ACT, Australia

**Keywords:** glycosaminoglycan, heparan sulfate, heparanase, hematopoiesis, homing, inflammation

## Abstract

Heparan sulfate (HS) is a highly acidic linear polysaccharide with a very variable structure. It is ubiquitously expressed on cell surfaces and in the extracellular matrix and basement membrane of mammalian tissues. Synthesized attached to various core proteins to form HS-proteoglycans, HS is capable of interacting with various polypeptides and exerting diverse functions. In fact, a bioinformatics analysis of mammalian proteins that express a heparin/HS-binding motif and are associated with the immune system identified 235 candidate proteins, the majority having an intracellular location. This simple analysis suggests that HS may, in fact, interact with many more components of the immune system than previously realized. Numerous studies have also directly demonstrated that HS plays multiple prominent functional roles in the immune system that are briefly reviewed in this article. In particular, the molecule has been shown to regulate leukocyte development, leukocyte migration, immune activation, and inflammatory processes.

## Introduction

**Figure 1:**
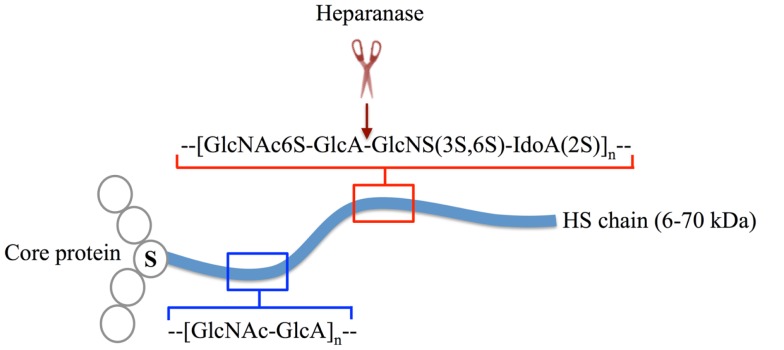
**The structure of HSPG**. HS chains (blue line) are linear polysaccharides composed of repeating disaccharide subunits, which in their unmodified form are d-glucosamine and d-glucuronic acid (blue box). During synthesis, HS chains are covalently attached to core proteins (open circles) at serine (S) residues. A single HSPG molecule may carry multiple HS chains or contain other glycosaminoglycans other than HS (not shown). HS modifications include various degrees of O and N-sulfation and epimerization of d-glucuronic acid to d-iduronic acid by HS-modifying enzymes (red box) (1). The modifications occur in regions (hot spots) along the polysaccharide chain, these hot spots being separated by regions of low sulfation. Post-synthesis structural alterations are primarily mediated by the endo-β-glucuronidase, heparanase, which cleaves HS chains within highly sulfated regions (cleavage site indicated by red scissors and arrow) (3).

Heparan sulfate (HS) is a glycosaminoglycan (GAG) that is ubiquitously expressed on cell surfaces and in the extracellular matrix (ECM) and basement membrane (BM). Each HS molecule is a linear polysaccharide composed of repeating disaccharides of hexuronic acid and d-glucosamine that can exhibit immense structural diversity due to substitution to varying extents with sulfate groups and epimerization of glucuronic acid to iduronic acid, with areas of high sulfation and glucuronic acid epimerization being co-located in “hot spots” throughout the molecule (Figure 1). HS is structurally related to heparin, an extremely highly sulfated form of HS that is restricted to mast cells. The biosynthesis and modification of HS chains is thought to take place within the endoplasmic reticulum, Golgi apparatus, and trans Golgi network, which in the end produce unique HS chains that are covalently attached to a range of core proteins to form HS-proteoglycans (HSPG) (Figure 1) (1, 2). After synthesis HS chains can be modified by the endoglycosidase, heparanase (3), and endosulfatases, Sulf1 and Sulf2 (4–6), to regulate HS availability and function. Although the core proteins can function independently of the HS chains they carry (7), HS predominantly dictates the ligand-binding capability and therefore the biological roles of HSPG (8). Furthermore, while different cell types may express similar core proteins, the HS chains these core proteins carry can be markedly distinctive, resulting in HSPG with highly diverse yet specialized roles in mammalian physiology (8, 9). In this mini-review, we will discuss some of the contributions of HS to the functioning of the immune system, notably leukocyte development, leukocyte migration, immune activation, and inflammatory processes.

## Different Cellular Locations of HS Chains

In general, cell surface HSPGs includes members of the transmembrane syndecans (syndecan-1-4) and glycosylphosphatidylinositol (GPI)-linked glypicans (glypican 1-6). ECM/BM associated HSPGs are comprised of perlecan, collagen type XVIII and agrin. These HSPGs are collectively termed “full-time” HSPGs. “Part-time” HSPG include cell surface CD44 (isoform 3 is HS-linked) and extracellular betaglycan, testican, and neuropilin (8, 10). Secretory vesicle-associated serglycin is a HSPG that is exclusively expressed intracellularly, particularly in mast cells (11). Furthermore, HSPG can also be localized in the nucleus where they potentially regulate gene transcription (12–16).

## Prevalence of HS-Binding Proteins in the Mammalian Immune System

Due to the structural similarities between HS and heparin, the latter is often used as an experimental model for biochemical studies of HS-protein interactions and predicting potential HS-binding partners. Several heparin-binding proteins are known to carry the “consensus” heparin/HS-binding motifs XBBXBX or XBBBXXBX (B being the basic amino acids arginine, lysine, or histidine and X being one of a range of aliphatic/aromatic amino acids) (17). If correctly displayed in the secondary structure and optimally positioned within the three-dimensional conformation of polypeptides, these sequences are hypothetically capable of facilitating strong ionic interactions with negatively charged GAGs (17, 18). Based on this simple amino acid sequence criterion, we screened for protein sequences matching selected G0 terms in the Ensembl database (release 72) with a custom Python script for murine gene products that carry these motifs and are listed on the UniProt database (www.uniprot.org) as being reported to have immunological functions. We identified a total of 235 HS-binding proteins in the mouse genome (Table 1), a list that includes known HS-binding proteins and many potential new ligands for HS. An intriguing feature of this analysis was that 66% of the molecules that potentially bind HS are expressed intracellularly, with only 18% being exclusively expressed on the cell surface and only 10% being in the extracellular compartment. Remarkably, only one HS-binding protein, the HS degrading enzyme heparanase, was identified that can be expressed in intracellular and extracellular compartments as well as being able to associate with plasma membranes. This finding is consistent with the multiple functional roles of the enzyme.

**Table 1:** **Hypothetical HS-interacting proteins**.

Cellular location	Total	Protein
Intracellular	154	Nlrp3, Nkap, Lrp5, Lyst, Lck, Irak4, Sla2, Mx2, Syk, Oasl2, Tcf3, Myo1e, Atp7a, Cplx2, Tusc2, Ak7, Vav3, Blnk, Spta1, Skap1, Fgr, Pmaip1, Aim2, Shb, Ddx60, Dapk3, Nfkb2, Apobec3, Ripk2, Nod1, Sptb, Oas1d, Mapk14, Ptbp3, March8, Dapk1, Zfp385a, Bcl2, Mecom, Chd7, Gpam, Ap3d1, Lcp2, Arid4a, Polr3a, Tnfaip1, Kat6a, Ung, Myo9b, Bcl6, Eprs, Sp3, Bag6, Plcg1, Nbn, Pml, Klf1, Rb1, Sfxn1, Bcl11a, Farp2, Jarid2, Xrcc6, Itk, Myo1f, Nlrc5, Cyp27b1, Ikzf1, Ddx58, Pip4k2a, Ciita, Foxj1, Rnf168, Prkdc, Pms2, Mll1, Stk3, Mef2c, Ahctf1, Prkx, Rag1, Batf3, Map3k14, Ilf2, Herc6, Card11, Card9, Dlg1, Itch, Dyrk3, Tnip3, Cdk6, Irgm1, Rnf31, Apc, Unc13d, Tlr13, Tlr8, Nedd4, Msh6, Pcid2, Sh2b2, Aicda, Myh9, Pik3cd, Zap70, Vav1, Stat5a, Tgtp1, Mx1, Enpp2, Dock2, Pgm3, Unc93b1, Plcg2, Stxbp2, Ifi44l, Zfp35, Inpp5d, Oas3, Cblb, Ostm1, Bcl11b, Eps8, Prkd1, Ctnnbl1, Polr3b, Samhd1, Nlrc4, Tec, Tet2, Map3k5, Pou1f1, Ncaph2, Stat6, Smarca4, Fnip1, Jak3, Cactin, Dicer1, Atm, Ikbkg, Satb1, Eif2ak2, Stap1, Msh3, Sgpl1, Cdk13, Foxe1, Zc3h8, Spib, Maea, March1, Ank1, Mink1
Plasma membrane	42	Mpzl2, Adam10, Ntrk1, Icosl, Cxcr5, Cd97, Tlr6, Pde2a, Adam9, Tlr1, Ccr3, Treml2, Tril, Tek, Lrrc8a, Il2ra, Selp, Fas, Hfe, Cd83, Cd22, Ccbp2, Ctla4, Tlr2, Klre1, Gpr183, Ccr7, Abcc9, Hoxb4, H2-M5, Thsd1, Dcstamp, Il7r, Procr, Amica1, Chrnb2, Tnfrsf13c, Csf1r, Tlr4, Tyr, Cd93, Eda, Cd40lg
Extracellular	24	Masp1, Ccl25, Osm, Il9, Bmp4, Inhba, Pdgfb, Scg2, Hc, Fam20c, Wnt2b, Lrrc17, Gas6, C7, Il1a, Wnt5a, Cxcl12, AI182371, C4b, Cxcl5, C8b, Ccl17, Serping1, Ccl28
Intracellular, plasma membrane, and extracellular	1	Hpse
Intracellular and plasma membrane	3	Blk, Flt1, Flt3
Intracellular and extracellular	2	Isg15, Prg4
Plasma membrane and extracellular	8	Tgfb1, Vegfa, Enpp3, Ctsg, C8a, Ptpro, Adam17, Enpp1

*List of murine proteins that carry “consensus” heparin/HS-binding motifs. The motifs adapted for bioinformatic screens are the standard motifs, which are primarily XBBBXXBX or XBBXBX (17) (http://gduserv.anu.edu.au/~cameron/protein.html). Short motifs are truncated versions of the standard motifs XBBBBX, BBXBB, or BBXXXBB (B = basic amino acids R, K, H; X = A, V, I, L, M, F, Y, W). Only proteins in the UniProt database (www.uniprot.org) that are known to play immunological roles are listed*.

Despite earlier reports claiming that HS negatively regulates gene transcription primarily by repressing the activity of p300 and pCAF histone acetyltransferase (14, 15), the bioinformatics screen implies that intracellular HS plays a more elaborate role in dictating cellular responses to various stimuli. Thus, it is predicted that HS interacts with several regulators of histone-modifying enzymes, such as Jarid2 (motif: MKRRHI), Kat6a (LHHLRM, KKVKK, RRVRK), and Mll1 (LRRFRA, IKKLRA, LKKAKA, VHRIRV, KKVKR, RHLKK) alongside key molecules that are involved in signal transduction and regulation of gene transcription, notably Vav1 (VKHIKI), STAT5A (KRIKR), STAT6 (KKIKR), Bcl6 (WKKYKF), Bcl11a (KHMKK), Ciita (LKRLKL), PTBP3 (VHRVKI, HRFKK), Lck (VKHYKI), IRAK1 (RRAKK), IRAK4 (HHIHR), Foxj1 (FKKRRL), Syk (RKAHH), ITK/TSK (IKHYHI), Card11 (KRFRK), Zap70 (KKLFLKR), Jak3 (IHKLKA, AKKLKF, RRIRR), and Cblb (RHFHH) and some components of the NF-κβ signaling pathway including NFKB2 (YHKMKI), IKBKG (MRKRHV), and Nkap (RRAKK, KKAKK, KKYKK). Interestingly, in the Rag1 protein, the presence of a HS-binding motif adjacent to a site critical for DNA binding (560D, UniProt) (AKRFRY), and overlapping the site that is essential for DNA hairpin formation [971F and 972R, UniProt (19)] (RRFRK), may imply a role for HS in regulating V(D)J recombination.

It is not surprising that cytokines including IL-1α (LKKRRL), IL9 (HRVKR), TGF-β1 (VKRKRI), and chemokines such as CCL17 (KHVKK), CCL19 (RRLKK), CCL25 (ARKRLVHM), CCL28 (VKRRRI), CXCL5 (KKAKR), and CXCL12 (VKHLKI) also carry HS-binding motifs, presumably allowing HS to act as their atypical receptors. Moreover, HS-binding motifs are also present on the cognate receptors for soluble factors such as the cytokine receptors IL-2Rα (HRWRK) and IL7R (KKVKH) and the chemokine receptors CCR3 (WKFFHA), CCR7 (AHRHRA), and CXCR5 (YRRRRL), indicating new roles for HS in regulating leukocyte homeostasis and trafficking. In addition, HS may regulate the availability of crucial components in the immunological synapse as indicated by the presence of HS-binding motifs in the leader sequences of ICOSL (WKKLHV) and CTLA4 (LRRYKA). The presence of HS-binding motifs in CD22 (KKARR) and CD40L (KKLKR) also suggests additional roles for HS in cell-cell communication and T cell costimulation.

Examination of the innate immune system reveals that, although HS is already known to interact with TLR-4 (20), it appears that HS may also be recognized by other TLRs, including cell surface TLR-1, TLR-2, and TLR-6, and endosomal TLR-8 and TLR13. To be more precise, the HS-binding motifs appear to be in the extracellular domains of TLR-4 (RHIFWRR) and TLR-2 (IRRLHA) and in the cytoplasmic domains of TLR-1 (HRARH) and TLR-6 (YHKLRA and HRARH). For the endosomal TLRs, both motifs (LKKLHL and LKKKHF) are facing luminal for TLR-8, and cytoplasmic (HRLRK) and luminal (LKRLKI) for TLR13. It is possible that HS is involved in regulating downstream signaling when the motifs are present in the TLR cytoplasmic domains. In contrast, when these motifs are facing the luminal or extracellular space, HS may be a ligand or a regulatory component that modulates the interaction between a TLR and its cognate ligands. Furthermore, HS-binding motifs are also present on inflammasome components such as Nlrp3 (LKKFKM), Nlrc4 (LKKMRL, RHIHR), and Aim2 (LKRFKY), implicating a role for HS in regulating the activation of inflammatory caspases. In another aspect of innate immunity, several studies have reported that heparin and HS are able to interact with several components of the complements system, including C1 (21), C1q, C1 inhibitor, C2, C4, C4b, C4bp, C6, C8, C9, Factor B, Factor D, Factor H (22), MASP1, MASP-2 (23), and complement receptors CR3 (CD11b/CD18) (24) and CR4 (CD11c/CD18) (25). In support of these observations, we have identified the presence of HS-binding motifs in complement proteins such as C4b (FRKFHL), Hc/complement C5 (FHKYKV), C7 (KRLYLKR), C8a (WRKLRY), and C8b (KRYRH) as well as regulatory components of the complement machinery including MASP-1 (KHWRR), Serping1 (HKIRK), and CD93/complement component C1q receptor (YHKRRA), further highlighting the role of HS as a major modulator of the complement system (26).

However, it should be noted that more in-depth analyses are required to assess the validity of these predicted interactions, in particular the demonstration that the predicted HS-binding sites are correctly presented within the secondary structure and final three-dimensional conformation of the putative HS-binding proteins. Also, there are known HS-binding proteins that lack the heparin/HS-binding motifs used in this analysis, suggesting that the list of binding proteins identified in this screen may, in fact, be an under estimate. Thus, at face value, the data set implies that a wide range of HS-binding proteins participate in the immune system.

## Functional Roles of HS in Immunity

Despite the previous section suggesting that there are many unknown HS-protein interactions that may control the immune system, there are a number of well-established functional roles for HS in immunity. Indeed HSPGs, through their HS chains, are involved in a broad spectrum of biological processes, profoundly influencing development (27), homeostasis (28), and the progression of many diseases (29). In the case of the immune system, HSPGs are fundamentally involved in regulating cell adhesion, cytokine and chemokine function, sensing tissue injury, and mediating inflammatory reactions. Each of these functional roles will be briefly discussed below, with specific examples given which highlight each function.

### Regulator of cell adhesion

Cell adhesion molecules are important to facilitate and regulate cell-cell signaling, migration and activation of leukocytes during development, homing, and recruitment to inflammatory sites. For example, of particular relevance to leukocytes development in the bone marrow is the receptor complex on hematopoietic stem cells (HSCs) comprised of CD45 and Mac-1 (CD11b/CD18) that has been shown to bind to surface HS on bone marrow-derived stromal cells and facilitate strong adhesion (30). In a related study, the HSPG glypican-3 enhances the antagonizing effect of tissue factor pathway-inhibitor (TFPI) on CD26, the stromal-bound ectopeptidase that is involved in cleaving surface CXCL12, a typical ligand for CXCR4 on HSC. As a result, glypican-3 indirectly supports the directional homing of grafted HSC toward, and their retention in, the bone marrow (31). Similarly, in the thymus a particular subset of cortical epithelial cells known as thymic nurse cells are reported to express high levels of highly sulfated HS that is thought to aid thymocyte adhesion and facilitate T cell development (32–35).

During an inflammatory response, HS positively regulates the recruitment of inflammatory cells at three different stages based on the following observations. First, endothelial surface HS can reduce neutrophil rolling velocity via L-selectin-mediated cell adhesion (36). Second, once attached, the HS-mediated Mac-1-CD44v3 interaction enhances the binding of leukocytes to the endothelial surface to drive extravasation (37). Finally, within the endothelial ECM/BM, collagen type XVIII promotes leukocytes infiltration in again an L-selectin-dependent manner (38, 39). However, the role of HS in the adhesion of leukocytes to the endothelium can occur paradoxically, in a biphasic manner. Under physiological conditions, glycocalyx HSPGs of pulmonary endothelial cells are known to impede neutrophil adhesion (40). Following the induction of an experimental sepsis model of acute lung injury, the localized production of TNF-α activates endothelial cells to produce heparanase, which in turn catalyzes the partial degradation of HS constituents of the glycocalyx. The loss of HS results in a significant increase in neutrophils binding (40), presumably via neutrophils L-selectin binding to residual endothelial HS and via cytokine-induced endothelial P- and E-selectin (41). Additionally, reduction (in wild-type mice) or deletion (in knockout mice) of syndecan-1 from murine endothelial cells strongly accentuates antigen-specific lymphocytes infiltration into inflammatory sites during a delayed-type hypersensitivity reaction (42).

### Modulator of cytokine and chemokine function

Soluble factors, such as cytokines and chemokines, are crucial to support growth, maintain homeostasis, and orchestrate immune cell trafficking across various locations. However, some of these molecules are inactive or susceptible to degradation in their native, soluble form. Furthermore, these factors need to be timely presented at the right site to exert their anticipated functions. HSPGs have been implicated in modulating various aspects of cytokine and chemokine function (43, 44).

HS-proteoglycans interact with various cytokines primarily on target cells or act as atypical cytokine receptors on cytokine presenting cell. The former situation enables HPSGs to regulate the availability and influence the interaction between cytokines and their cognate receptors on target cells. For example, the binding of cell surface HS to cytokines such as IL-7 and IFN-γ proved critical to protect them against proteolysis (45, 46). Furthermore, the lack of HSPG expression on the mouse pro-B cell surface severely impairs IL-7-dependent maturation toward pre-B cells suggesting that in this situation HS acts as a primary IL-7 receptor. In addition, HSPG can contribute to IL-7 biological activity by presenting IL-7 on stromal cells to promote lymphopoiesis in the bone marrow (47). In the thymus, however, the ability of stromal cell HSPGs to bind IL-7 and aid thymocytes development is dispensable (48). Depending on the degree of sulfation, cell surface HSPG has also been shown to potentiate the IFN-γ-IFN-γ-receptor interaction (49). Importantly, HSPG also facilitate cytokine localization in specific niches, forming depots where they can be made available to target cells. For instance, perlecan binds IL-2, sequestering it from the circulation and subsequently depositing the cytokine in the marginal zone and red pulp of the murine spleen to modulate murine T cells homeostasis (50). HSPGs also facilitate the storing of IL-2 within the vascular smooth muscle wall where cytokine availability is regulated through heparanase-mediated ECM degradation (51).

The ability to bind and present a chemokine to target cells is insufficient to drive cell migration, the hallmark of chemokine function. HSPGs are not only capable of binding and assisting in inducing conformational changes in bound chemokines (52, 53), but also contribute to the establishment of immobilized (haptotactic) chemokine gradients in tissues (54). For example, HSPG facilitate the oligomerization of bound RANTES/CCL5, CXCL8, MCP-1, and MIP-1, thereby allowing better recognition by their cognate G-protein-linked transmembrane receptors (55). In addition to the sequestration of CXCL2 (56), HSPG also mediate transcytosis of CXCL8 across the endothelium, presenting both chemokines on the luminal side and establishing haptotactic gradients that aid neutrophils recruitment during inflammation (36). In a separate study, the migration of tissue dendritic cells (DC) to regional lymph nodes and the local positioning of DC within lymph nodes was also found to be mediated by a HS-dependent haptotactic gradient of CCL21 (57). A similar interaction is believed to facilitate lymphocytes homing through the high endothelial venules into peripheral lymphoid organs (58, 59), although it is unclear if HS also influences local positioning in specific niches. Also, shedding of HSPG, such as syndecan-1 (60) and removal of glucosamine 6-*O*-sulfate by the endosulfatase, Sulf2 (6) has been implicated in regulating the interaction between HS and various chemokines. Furthermore, the inactivation of HS-modifying enzymes can modify neutrophil binding to the endothelium, Ndst1 (HS *N*-deacetylase/*N*-sulfotransferase) deletion severely impairing (36), while Hs2st (HS 2-*O*-sulfotransferase) deletion significantly augmenting (61), neutrophil binding. These studies support the concept of regulating HS function by altering the availability of enzymes that are involved in HS biosynthesis.

### A sensor of tissue injury

Tissue injury may induce cell necrosis, an event that is often associated with the release of various endogenous damage-associated molecular pattern (DAMP)-containing molecules that are potent inducers of inflammatory responses and initiators of tissue repair mechanisms (62). Both surface bound HSPG and soluble HS participate in sensing tissue injury and also in repair mechanisms. For example, endothelial cell surface HS mediates the oligomerization of the receptor for advanced glycation endproducts (RAGE) (63) and together form a receptor complex that efficiently recognizes the chromatin protein, high-mobility group protein B1 (HMGB1) released from necrotic cells (64). As part of the tissue repair mechanism, HS on the surface of professional phagocytes also assists in the clearance of necrotic cells (65). In fact, soluble HS itself can also function as a DAMP (66) by interacting with TLR-4 on leukocytes (20). This interaction has been shown to modulate the release of pro-inflammatory cytokines by macrophages (67) and markedly induce the maturation of DC, as indicated by the up-regulation of MHC-II, CD40, ICAM-1, CD80, CD86, and reduced antigen uptake, a typical phenotype of a professional antigen presenting cells (68). Although this is beneficial in triggering immune activation following an insult (20), it is also implicated in the underlying mechanism of disease progression which can occur in experimental pancreatitis (69), sepsis-like syndrome (70), hyperacute rejection in graft-versus-host disease (GvHD) (71), and cardiac injury (72).

### Physical barrier to leukocyte migration

The ECM/BM associated HS is crucial to form a temporary depot of HS-binding soluble factors and to form a physical barrier that supports tissue integrity. In order to migrate, particularly through blood vessel walls, leukocytes need to break down the ECM/BM barrier and heparanase is primarily involved in this process (10, 73). For example, tissue DCs increase the availability of cell surface heparanase to aid ECM degradation before migrating into lymphatic vessels leading toward regional lymph nodes where they induce antigen-specific responses (74). Subsequent studies have suggested that the matrix metalloproteinase, MMP-14, cooperatively works with heparanase to more efficiently degrade ECM/BM barriers (75). During inflammation, infiltrating monocytes and neutrophils also exhibit similar modes of degrading ECM/BM barriers to aid their extravasation (76, 77). It has also been demonstrated that heparanase derived from infiltrating leukocytes is primarily responsible for the destruction of the pancreatic islet β-cells that produce insulin, thereby providing a novel explanation for the underlying immunopathology of autoimmune Type 1 diabetes (78). In a separate study, leukocytes were shown to also use endogenous myeloperoxidase to produce oxidants that degrade the core protein of perlecan, releasing soluble factors and allowing leukocyte migration across ECM/BM barriers (79). Although HS can also be degraded by nitric oxide (80, 81) and reactive oxygen species (82–84), their direct relevance in the degradation of ECM/BM-associated HSPGs and therefore their contribution to leukocytes extravasation is yet to be elucidated.

## Concluding Remarks

The evidence presented in this mini-review further corroborates the fundamental importance of HS in the mammalian immune system. HSPGs, primarily through their HS side chains, regulate various aspects of the immune system ranging from hematopoiesis to homing of leukocytes to peripheral tissues and, most importantly, regulating the elicitation of immune responses. Perturbing HS function or availability has been proven to results in various abnormal immune phenotypes. Furthermore, based on a simple bioinformatics screen presented in this review, it is suggested that HS may in fact interact with many more components of the immune system than previously realized. A better understanding of HS function across various systems is fundamental to exploit its potential in boosting beneficial immune responses and also in finding treatments for related immunopathologies.

## Conflict of Interest Statement

The authors declare that the research was conducted in the absence of any commercial or financial relationships that could be construed as a potential conflict of interest.
